# Trends in availability and prices of subsidized ACT over the first year of the AMFm: evidence from remote regions of Tanzania

**DOI:** 10.1186/1475-2875-11-299

**Published:** 2012-08-28

**Authors:** Prashant Yadav, Jessica L Cohen, Sarah Alphs, Jean Arkedis, Peter S Larson, Julius Massaga, Oliver Sabot

**Affiliations:** 1 The William Davidson Institute, University of Michigan, 724 E University Avenue, Ann Arbor, MI, 48109, USA; 2 Ross School of Business, University of Michigan, 701 Tappan Avenue, Ann Arbor, MI, 48109, USA; 3 School of Public Health, University of Michigan, 1415 Washington Heights, Ann Arbor, MI, 48109, USA; 4 Harvard School of Public Health, 677 Huntington Avenue, Boston, MA, 02115, USA; 5 Results for Development Institute, 1100 15th Street, NW, Suite 400, Washington, DC, S, USA; 6 National Institute for Medical Research, P.O. Box 9653, Dar es Salaam, Tanzania; 7 Clinton Health Access Initiative, 383 Dorchester Avenue, Suite 400, Boston, MA, 02127, USA

**Keywords:** Malaria treatment, ACT, Anti-malarial subsidy, AMFm, Remote availability, Drug shops

## Abstract

**Background:**

The Affordable Medicines Facility for malaria (AMFm) is a pilot supra-national subsidy program that aims to increase access and affordability of artemisinin combination therapy (ACT) in public sector clinics and private retail shops. It is unclear to what extent the AMFm model will translate into wide scale availability and price reductions in ACT, particularly for rural, remote areas where disparities in access to medicines often exist. This study is the first to rigorously examine the availability and price of subsidized ACT during the first year of the AMFm, measured through retail audits in remote regions of Tanzania.

**Methods:**

Periodic retail audits of Accredited Drug Dispensing Outlets (ADDOs) were conducted in two remote regions of Tanzania (Mtwara and Rukwa). Temporal and spatial variation in ACT availability and pricing were explored. A composite measure of ADDO remoteness, using variables, such as distance to suppliers and towns, altitude and population density, was used to explore whether ACT availability and price vary systematically with remoteness.

**Results:**

Between February 2011 and January 2012, the fraction of ADDOs stocking AMFm-ACT increased from 25% to 88% in Mtwara and from 3% to 62% in Rukwa. Availability was widespread, though diffusion throughout the region was achieved more quickly in Mtwara. No significant relationship was found between ACT availability and remoteness. Adult doses of AMFm-ACT were much more widely available than any other age/weight band. Average prices fell from 1529 TZS (1.03 USD) to 1272 TZS (0.81 USD) over the study period, with prices in Rukwa higher than Mtwara. The government recommended retail price for AMFm- ACT is 1,000 TZS ($0.64 USD). The median retail ACT price in the final round of data collection was 1,000 TZS.

**Conclusions:**

The AMFm led to large increases in availability of low priced ACT in Tanzania, with no significant variation in availability based on remoteness. Availability did remain lower and prices remained higher in Rukwa, which is a more remote region overall. Low availability of child and adolescent ACT doses could be due in part to lower quantities of non-adult packs imported into Tanzania. Future research will explore whether increased availability and affordability persists and whether it translates into higher ACT use in Tanzania.

## Background

In malaria endemic settings, the private retail sector constitutes a major source of treatment seeking for fever. Although people often seek treatment at multiple outlets, 40-60% of caregivers of children in many countries first seek treatment in drug shops and pharmacies [1,2]. Moreover, a high proportion of individuals who only seek treatment in one place, rely on private retail outlets [1]. However, as a number of studies have shown, artemisinin-based combination therapy (ACT), the first-line anti-malarial recommended in most malaria endemic countries, is often unavailable and unaffordable from sources outside of public health providers. A recent six-country study found that less than 25% of private sector shops stocked at least one quality-assured ACT, compared to 43-85% of public or not-for-profit facilities, and that ACT was 5–24 times more expensive than non-artemisinin therapy [3]. A number of other studies have highlighted similar problems [4,5].

The Affordable Medicines Facility for Malaria (AMFm) is a pilot program, hosted by the Global Fund to Fight AIDS, Tuberculosis and Malaria and launched in July 2009, that negotiates prices with WHO prequalified ACT manufacturers and then allows pre-approved private and public sector importers to place orders for subsidized ACT. This program is currently being piloted in seven African countries including the United Republic of Tanzania [mainland and Zanzibar]. The goals of the AMFm are to increase the availability and reduce the price of ACT to levels similar to that of less effective anti-malarials (such as sulphadoxine-pyrimethamine and chloroquine), and to displace artemisinin monotherapy whose widespread availability and improper use threaten to accelerate parasite resistance to artemisinin [6].

A number of pilots of ACT subsidies in recent years in Tanzania, Kenya, Cambodia, and Senegal demonstrated an increase in availability and decrease in prices of ACT in retail outlets [4,5,7], as a result of the subsidy. However, a key question remained as to whether the results of these smaller scale regional or national pilots would also be realized under the multi-country national scale subsidy model embodied in the AMFm. Moreover, some of these studies showed higher availability of subsidized ACT in urban outlets compared to outlets in remote, rural areas [5,8], which raised questions about the ability of such a subsidy to successfully reach the poorest and most vulnerable communities. Cohen *et al.* did a rigorous evaluation of the ACT subsidy pilot in Tanzania over a 15 month period and found that considerable geographical disparities persisted in the availability of ACT, with shops located closer to district towns and major roads more likely to stock subsidized ACT [9].

The Government of Tanzania and other stakeholders also were keen to understand how equitable would be the access to subsidized ACT as a result of the AMFm. As a result, Tanzania’s AMFm application to the Global Fund included a description of a research study with the aim of understanding the socio-economic and spatial variation in the availability of AMFm-subsidized ACT and testing whether a supply-side incentive could increase availability of subsidized ACT in remote shops. The results reported here represent the early results from this work. The early results suggested that a supply side incentive was not necessarily required and thus the study continued in an observational and monitoring capacity only.

This study monitored the availability and price of AMFm-subsidized ACT in two remote regions of Tanzania, Rukwa and Mtwara, through data collection in a census of licensed drug shops called Accredited Drug Dispensing Outlets (ADDOs). Recognizing that drug shops are an important source of anti-malarial treatment in Tanzania, but are staffed by unqualified shopkeepers and operated in poor conditions, the Tanzanian Food and Drug Administration (TFDA) in 2002 created a new class of drug shops known as accredited drug dispensing outlets (ADDOs). Existing drug shops were upgraded to ADDOs through a combination of dispenser training, financial incentives, accreditation and regulation [10,11]. See Additional file 1 for details on the training, education and dispensing practices at the ADDOs in the study.

The study aimed to answer a number of key questions: (1) How did availability and price of ACT change over the first 12 months of the AMFm in these remote regions?; (2) Within these regions, did availability and price differ among shops that were relatively more remote compared to more urban shops?

## Methods

### Region selection

**Figure 1:**
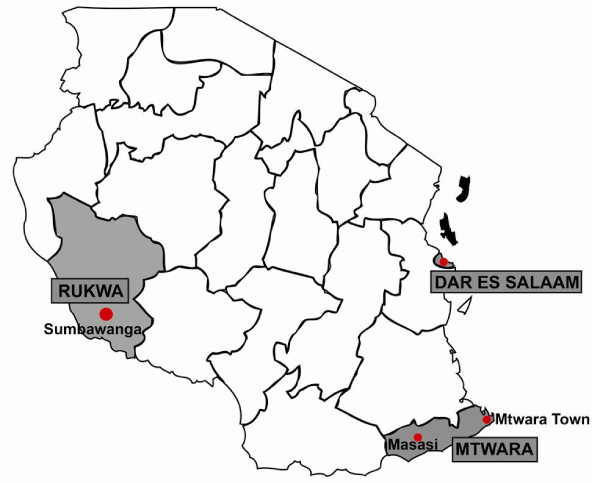
Map of two study regions selected and the major towns in each region.

In consultation with Tanzania’s National Malaria Control Program (NMCP), two regions in Tanzania were selected for inclusion in the study based on the following criteria: moderate to high malaria endemicity, overall remoteness of the region, lack of other malaria interventions which could confound interpretation of the impact of the AMFm, and the total number of ADDOs in the region (enough to provide an adequate sample size for the study). The selected regions were Mtwara and Rukwa (Figure 1). Mtwara is located along the southeastern coast of Tanzania bordering Mozambique on its southern border. Rukwa is located in south-western Tanzania between Lake Tanganyika and Lake Rukwa. Rukwa is among the largest regions in Tanzania (it is approximately four times the size of Mtwara), and also has one of the lowest population densities with 17 people per square kilometer, as compared to Mtwara with 67 people per square kilometer. Both regions are predominantly rural with the majority of the population engaged in agriculture and livestock husbandry as their economic mainstay. However, the regions have varying geography, transport and trade linkages and malaria ecology [12-14]. The distance between Sumbawanga, the main urban center in Rukwa, and Dar es Salaam (1,150 km) is more than twice the distance between Mtwara’s main urban center and Dar es Salaam (556 km) [15]. The roads between Dar es Salaam and Mtwara town are better-developed and there are daily, direct commercial flights operating between the regions. The transport methods to Rukwa are more challenging, with poorer road conditions and limited commercial flights, which just recently began operation.

Estimated malaria prevalence among children ages 6–59 months in Mtwara and Rukwa was 33.6%, and 11% respectively in the 2007–2008 Tanzania Malaria Indicator Survey [12]. The use of mosquito nets by children under 5 was 85.4% (n = 226) in Mtwara compared to 78.85% in Rukwa (n = 218) [12]. Among the children under the age of 5 with reported fever, the percentage seeking advice from a health facility or provider was 84.9% (n = 228) in Mtwara and 68.5% (n = 271) in Rukwa. Additionally, more children under the age of 5 in Mtwara with fever were likely to take an anti-malarial, with approximately 83.5% of children reported to have taken an anti-malarial in Mtwara compared to 63.4% in Rukwa [12]. The first-line treatment for uncomplicated malaria in Tanzania is artemisinin-based combination therapy (ACT)— specifically artemether-lumefantrine is used in the public sector. Other ACT formulations such as Arestunate-Amodiaquine (As-AQ) are less frequently used in the private sector drug shops.

Mtwara has four pharmaceutical wholesalers and Rukwa has three. In Mtwara, two are located in Mtwara Town and two in Masasi Town, while all three wholesalers in Rukwa are located in Sumbawanga Town. There are 146 ADDOs in Mtwara as compared to the 306 ADDOs in Rukwa. The average distance from an ADDO in Mtwara to Dar es Salaam is 440 km compared to the 868 km in Rukwa.

### Drug shop selection

This study required a retail audit to capture the stock of anti-malarial medicines and their prices at Accredited Drug Dispensing Outlets (ADDOs), the lowest level of drug shops that currently have approval from the Tanzania Food and Drug Authority (TFDA) to sell ACT. ACT can also be distributed in licensed private sector pharmacies, as well as public and private sector dispensaries, public health centers, and district and regional hospitals. In August 2010, the study team used government records from the TFDA and district health offices to conduct a comprehensive ADDO census to verify and record GPS coordinates. ADDOs identified during this process were included in the study only if verbal consent was obtained by the drug shop owner or attendant on the day of the visit. Over the course of the five survey rounds, over 97% of surveyed ADDOs in Mtwara and 99% of ADDOs in Rukwa region provided consent. The same ADDOs were repeatedly visited throughout the course of the study.

### Retail survey method

A retail survey tool was developed to record information on anti-malarial drugs in stock on the day of the survey, prices for each of the anti-malarials stocked, numbers and qualifications of staff, sources of supply for anti-malarials and other medicines, and other shop level attributes. A national list of all registered anti-malarial drugs was obtained from the TFDA to develop the list of anti-malarials and other medicines to be included in the survey tool. Training manuals were developed prior to the start of the retail surveys to explain the questionnaire and provide details on active ingredients, dosing and pack sizes of the anti-malarials registered in Tanzania. The training manuals included images/pictures of commonly used anti-malarials to assist the surveyors in identifying the drugs stocked.

The first round of retail audits commenced in mid February 2011, three months after the first order of AMFm-ACT arrived in Tanzania. The final round reported in this study was conducted in January 2012, though the study continued till June 2012 Figure 2). Surveyors showed up at the ADDOs unannounced in all rounds, but collected phone numbers during the first round for follow-up only if the ADDO owner or attendant was not available at the time of the visit. This analysis contains five rounds of retail survey data (February 2011-January 2012) from Mtwara and Rukwa regions. There were two types of surveys. The basic retail survey included a brief structured interview with the dispenser, as well as an inspection of all anti-malarial inventory in stock on the day of the visit. The comprehensive retail survey included questions about stocking practices, knowledge of appropriate malaria treatment, and the dispenser’s training. Of the five rounds of retail surveys analyzed in this study, the first round was a comprehensive survey and the following four rounds were basic retail surveys.

**Figure 2:**
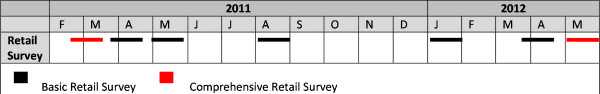
Retail Audit Schedule (this study is based on data collected till Feb 2012).

### Ethical approval

The study was approved by the Institutional Review Board of Harvard University (Protocol #19372-102) and the National Institute of Medical Research of Tanzania (NIMR/HQ /R.8a/Vol. IX/1017).

### Data collection and analysis

The study team used Surveybe software to develop electronic survey tools used for all data collection activities. Data were exported from the surveyor’s Ultra Mobile Personal Computers (UMPCs), which hosted the tools, and were aggregated on a daily basis while data collection activities were ongoing. The data collected was exported to STATA 10 (STATA Corporation, College Station, TX, USA) for statistical analysis.

### Definition of AMFm-ACT availability and price

The ADDO owner or attendant was asked to show all anti-malarial brands and pack sizes available in the shop on the day of the retail survey. AMFm-ACT are manufactured by one of the AMFm eligible manufacturers (Ajanta, Cipla, Guilin, Ipca, Novartis, Sanofi-Aventis, Quality Chemical Industries) and are additionally identifiable by the presence of the ACT leaf logo on the drug packaging. Availability was determined by the presence of at least one unit of any pack size of an AMFm-ACT at the ADDO on the day of the visit. See Additional file 2 for the availability of non-subsidized ACT manufactured by WHO prequalified manufacturers.

All pack sizes of subsidized ACT were recorded and the price was determined by asking the following question in reference to a specific pack size of AMFm-subsidized: “*How much does one unit cost the individual customer*?”

### Spatial/temporal patterns

Temporal patterns of ACT stocking in ADDOs were illustrated using descriptive and graphical methods. Spatial patterns of ACT stocking were identified using the recorded GPS locations of ADDOs. Chloropleth maps of ACT stocking (colour/shading intensity of a geographical area in proportion to ACT stocking in that area) in ADDOs were produced using the non-parametric inverse distance weighting interpolation method.

### Associations of remoteness with subsidized ACT stocking

Characteristics of the area surrounding each ADDO were recorded in order to determine associations of factors such as proximity to major sources of drug stocks and remoteness with ACT stocking patterns. For each ADDO, the distance to the reported supplier of anti-malarials and the distance to the nearest major town were estimated using a geographic information system (GIS) layer of major roadways in Tanzania and the Network Analyst tool in ArcGIS. Altitude of each ADDO was extracted from an elevation raster of Tanzania and added to the database. To estimate the likely catchment areas of ADDOs, a population roster of Tanzania was used (2011). A 5 km radius was drawn around each ADDO, and the total population within that radius was summed and added to the master database. Associations of spatial covariates such as distance to reported wholesale supplier and distance to major towns with ACT stocking were tested using standard T-tests for differences of means. Each covariate was tested individually using a significance level of .05. Spatial associations were tested separately for Rukwa and Mtwara.

A composite measure of remoteness was then created using Principal Components Analysis methodology, including the distance to supplier, distance to major town, altitude, population and the standard categorical accessibility measure reported by the survey teams. The first principal component was taken to be a continuous measure of remoteness, and divided into quintiles. The results of the PCA were observed and a measure of remoteness was assigned using an appropriate clustering of PC quantiles.

The final PCA-based measure of remoteness included all available spatial variables: distance to supplier, distance to region-specific major towns, subjective road quality classifications assigned by the survey teams, altitude of ADDO and population in the area surrounding the ADDO. Distance to supplier, fair and poor road access, were all assigned negative weights, whereas altitude, population and good road access were assigned positive weights. The composite measure was used as a graded measure of remoteness, with high (positive) values representing more “accessible” shops and low (negative) values representing “remote” shops. The measure was divided into quintiles. The first three quintiles were taken to be “remote” areas while the top two were assumed to be “non-remote” areas.

## Results

### Anti-malarial sourcing

**Figure 3:**
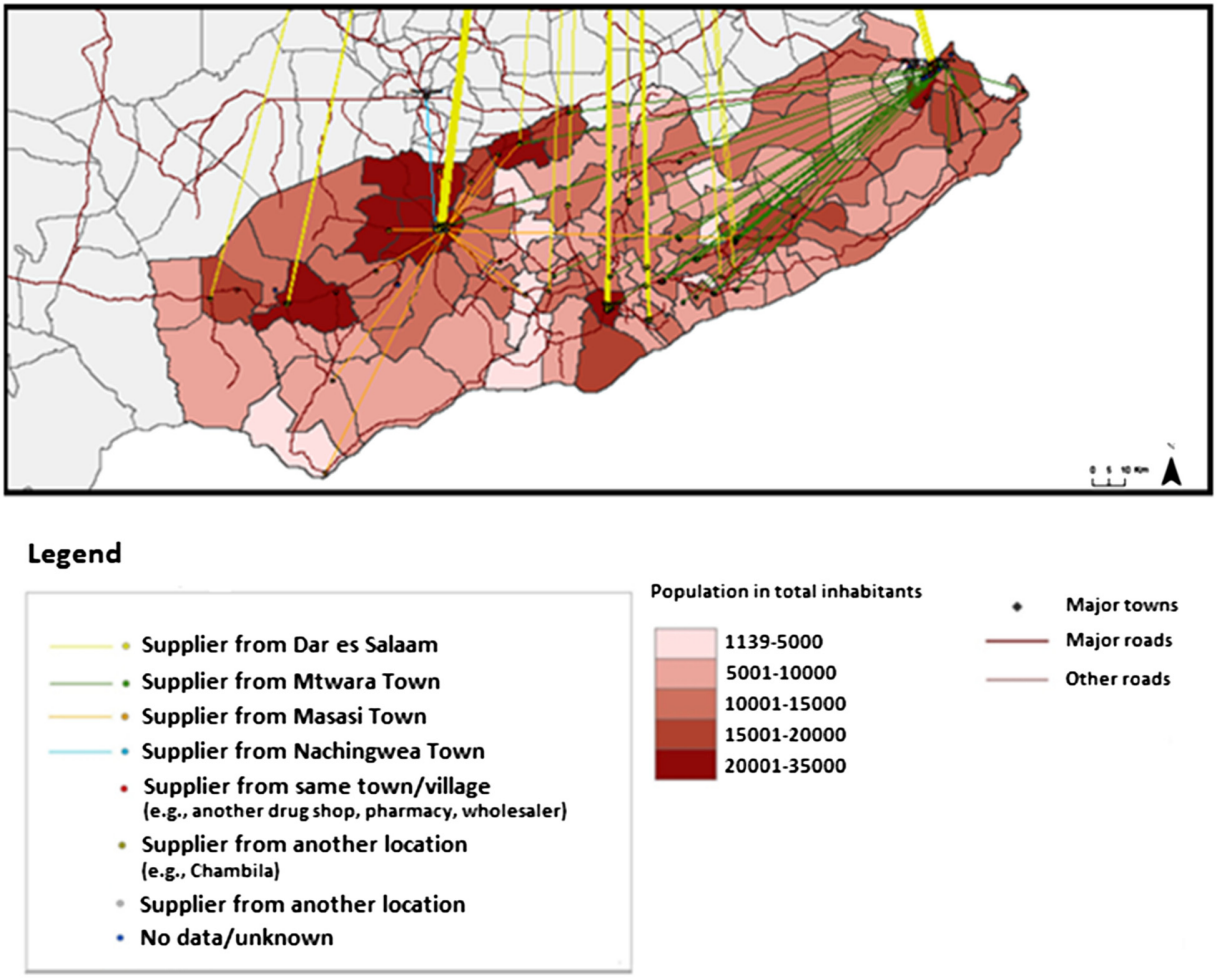
Anti-malarial sourcing by ADDOs in Mtwara.

**Figure 4:**
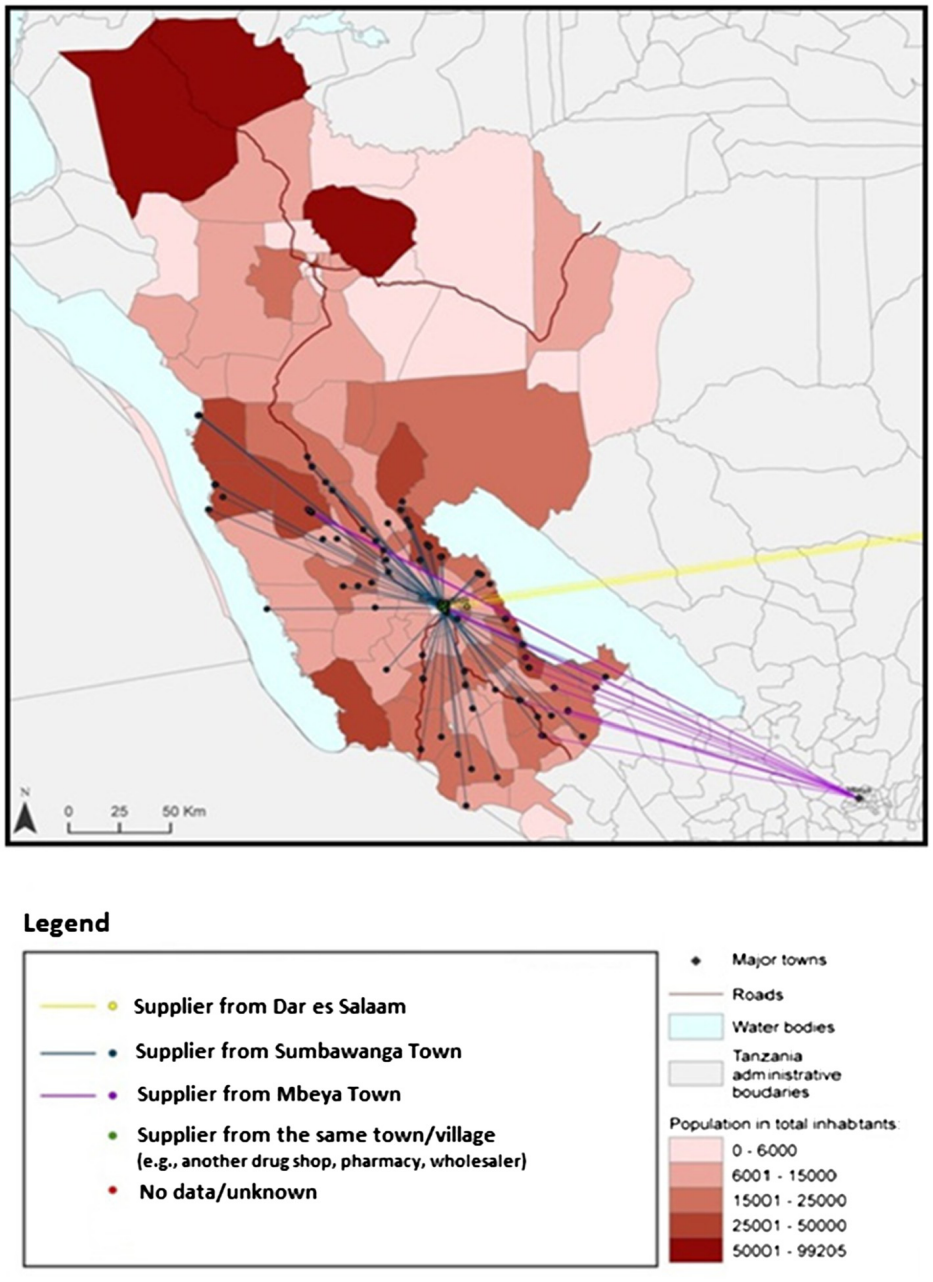
Anti-malarial sourcing by ADDOs in Rukwa.

89.58% of the ADDOs surveyed obtained their stock of anti-malarials by travelling to the wholesaler or source of supply, while less than 8% reported being able to place an order by phone and receive supplies directly at their shop. 50% of retail outlets surveyed in Mtwara obtained their anti-malarial medicines from Mtwara town, 32% from Dar es Salaam, and the rest from other towns in the region. In Rukwa, 64% of the retail outlets surveyed obtained their anti-malarials from Sumbawanga town, 14% from Mbeya town in the Mbeya region, 3% from Dar es Salaam and the rest from other towns in the region (Figures 3 and 4).

### Availability

**Figure 5:**
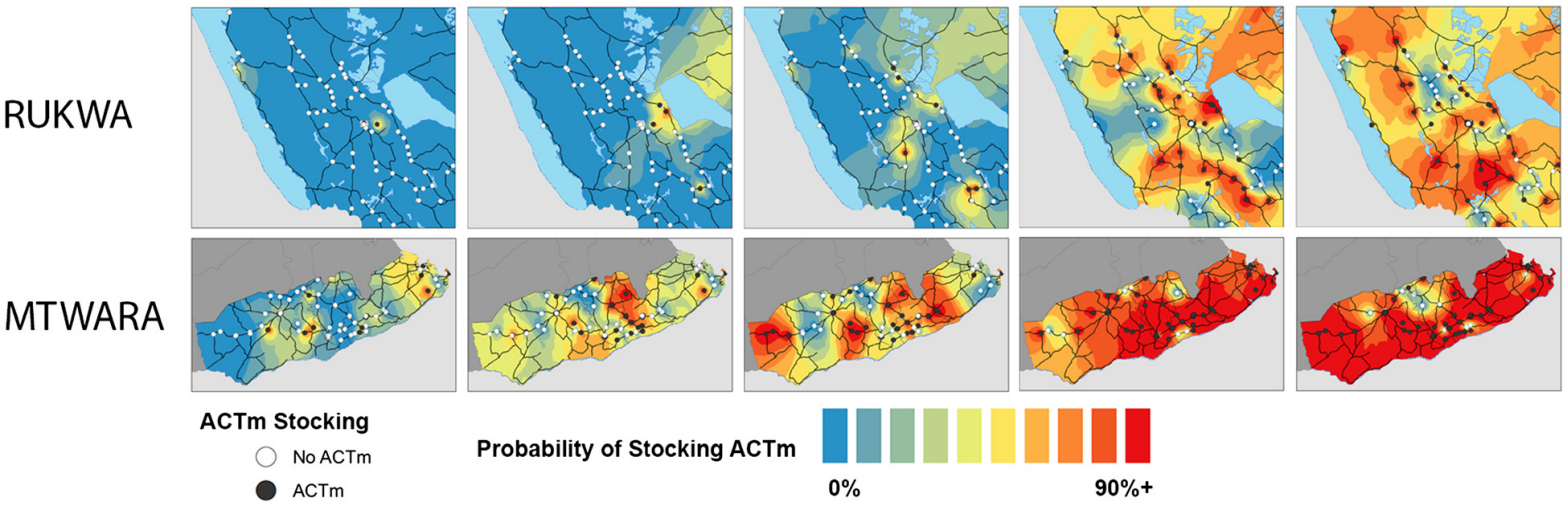
Spatial distribution of AMFm-ACT stocking over five rounds of surveys in Rukwa and Mtwara.

Stocking of AMFm subsidized ACT increased over time in both Rukwa and Mtwara, though stocking levels in Mtwara were higher overall (Table 1). By the fourth survey round, 88% of all shops in Mtwara were stocking AMFm subsidized ACT (Table 1). Stocking levels in Rukwa, while significantly lower than that of Mtwara in every round, continued to rise up until the most recent survey round at over 62% (Table 1) and grew at a faster rate than Mtwara. In Mtwara, stocking of AMFm subsidized ACT was spatially widespread by the second and third survey rounds (Figure 5). Spatial patterns of AMFm-subsidized ACT stocking in Rukwa appear to be initially concentrated in areas adjacent to Lake Rukwa, and later in the more urbanized areas of Sumbawanga and Mbeya (Figure 5); though by round 5, nearly every area of the region has at least one shop which stocks AMFm subsidized ACT.

**Table 1:** Percentage of shops stocking AMFm-subsidized ACT by region and survey round with tests for differences in proportions

	**Overall**		**Mtwara**		**Rukwa**		**T Test for difference between Mtwara and Rukwa**
	**N**	**Percent**	**N**	**Percent**	**N**	**Percent**	**P**
**R1: mid Feb 2011**	255	12.55%	110	24.55%	145	3.45%	<.0001
**R2: Apr 2011**	253	26.09%	109	50.46%	144	7.64%	<.0001
**R3: May 2011**	237	37.55%	102	61.76%	135	19.26%	<.0001
**R4: Aug 2011**	234	66.67%	97	87.63%	137	51.82%	<.0001
**R5: Jan 2012**	243	73.25%	102	88.24%	141	62.41%	<.0001

More ADDOs were stocking adult packs of AMFm subsidized ACT across all rounds compared to child packs (Table 2). Availability of child packs by round five was still low, with less than 30% of ADDOs stocking the lowest dose appropriate for children under 3 years (ACT pack size 6x1) by the last round of data collection (Table 2). There were very few observations of subsidized artesunate + amodiaquine (ASAQ) across all rounds, with less than 0.41% of ADDOs stocking an adult or child pack of this drug by round 5.

**Table 2:** Overall availability of AMFm-subsidized ACT by drug type, pack size and round

**Drug Type**	**Form**	**AMFm-subsidized pack size**	**Round 1 (n = 255)**	**Round 2 (n = 253)**	**Round 3 (n = 237)**	**Round 4 (n = 234)**	**Round 5 (n = 243)**
AL	FDC	6x 1	1.18%	5.14%	4.22%	14.53%	27.57%
	FDC	6x2	0.78%	3.16%	5.06%	10.26%	24.28%
	FDC	6x3	0%	2.37%	3.80%	6.84%	22.63%
	FDC	6x4	10.98%	22.53%	33.33%	56.41%	55.14%
ASAQ	FDC	3x1 (25 mg/67.5 mg)	--	--	--	--	0.41%
	FDC	3x1 (100 mg/270 mg)	--	--	--	1.28%	0.41%
	FDC	3x2 (100 mg/270 mg)	--	--	--	0.43%	--
	Co-Blist	12 + 12	--	--	--	--	0.41%

The availability of non-subsidized ACT manufactured by WHO prequalified ACT manufacturers was negligible and almost all the WHO prequalified ACT found in the ADDOs was AMFm-subisidzed ACT. See Additional file 2 for details on the availability of non-subsidized ACT manufactured by WHO prequalified ACT manufacturers.

### Differences in availability by ADDO remoteness

**Figure 6:**
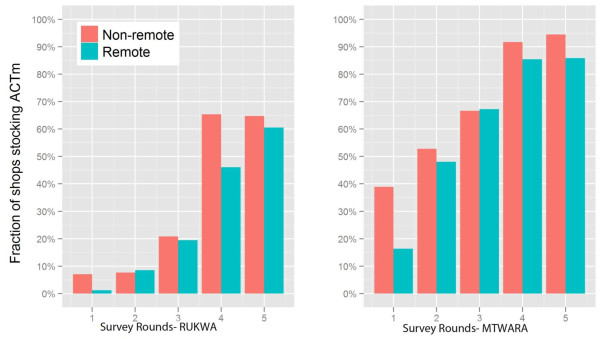
AMFm-ACT Stocking vs. Shop Remoteness in Rukwa and Mtwara.

Figure 6 shows temporal patterns of ACT stocking for remote and non-remote shops. Though an increasing percentage of shops stocked ACT over the five survey rounds, in most of the survey rounds the percentage of remote shops stocking ACT was lower than that of non-remote shops in both regions. This difference was wide in the first round but very small through the rest of the study period, other than round 4 in Rukwa.

### Pricing

The government recommended retail price for an adult dose of AMFm-subsidized ACT is 1,000 TZS (0.64 USD) and has been promoted widely on television and radio spots throughout Tanzania. Starting in July/Aug 2011, this recommended price was added to printed materials for promoting AMFm-subsidized ACT. It is important to note that prices for AMFm-subsidized ACT can only be reported for shops in which these medicines were stocked and, thus, the number of price observations for Rukwa in early rounds is small and inference limited.

The mean reported price of an adult dose of AMFm subsidized artemether-lumefantrine (AL) notably decreased between Round 1 and Round 5 (Table 3) with prices remaining somewhat stable between Round 1-Round 3 (Feb-May 2011) at roughly 1,530-1,580 TZS ($1.03-$1.04 USD), then decreasing to a mean of 1299 TZS ($0.81 USD) by Round 4 (Aug 2011) (See Additional file 3).

**Table 3:** Retail Price of AMFm-ACT (Reported by Shopkeeper)

**Region**	**Price for an adult dose of AL**	**Round 1: February 2011**				**Round 5: January 2012**			
		**Median retail price**	**Mean retail price**	**Min**	**Max**	**Median retail price**	**Mean retail price**	**Min**	**Max**
**Overall**	Price (TZS)	1500	1529	1000	3000	1000	1272	800	3000
(n1 = 28, n5 = 143)	[Price (USD)]	$1.01	$1.03	$0.67	$2.01	$0.64	$0.81	$0.51	$1.91
**Mtwara**	Price (TZS)	1500	1480	1000	3000	1000	1106	800	2000
(n1 = 25 , n5 = 78)	Price (USD)	$1.01	$0.99	$0.67	$2.01	$0.64	$0.71	$0.51	$1.28
**Rukwa**	Price (TZS)	1500	1933	1300	3000	1500	1470	1000	3000
(n1 = 3, n5 = 65)	Price (USD)	$1.01	$1.30	$0.87	$2.01	$0.96	$0.94	$0.64	$1.91

By round 5, the reported median price for an adult dose of AL in Rukwa was 1,500 TZS ($0.96 USD), 50% higher than the median reported price in Mtwara at 1,000 TZS ($0.64 USD) (Table 3). The difference in the mean prices of adult packs in Rukwa and Mtwara is significant in Rounds 4 and 5 (Table 4). No significant differences were observed in adult AL prices between remote and non-remote ADDOs (Table 5).

**Table 4:** Mean pricing (in TSh) of AMFm-ACT (AL-6x4 packs only) by region with tests for differences

	**Overall**	**Mtwara**		**Rukwa**		**T Test for differences**
	**Price**	**N**	**Price**	**N**	**Price**	**P**
**R1: mid Feb 2011**	1529	25	1480	3	1933	0.488
**R2: Apr 2011**	1578	50	1564	8	1662.5	0.471
**R3: May 2011**	1580	54	1530	26	1685	0.049
**R4: Aug 2011**	1299	76	1155	63	1471	<.0001
**R5: Jan 2012**	1272	77	1108	66	1463	<.0001

**Table 5:** AMFm-ACT: Price and Remoteness

	**Rukwa**					**Mtwara**				
	**Remote**		**Non-Remote**		**T test**	**Remote**		**Non-Remote**		**T test**
	**N**	**Price**	**N**	**Price**	**p**	**N**	**Price**	**N**	**Price**	**p**
**R1: mid Feb 2011**	0	NA	3	1933	0.27	8	1687	14	1429	0.27
**R2: Apr 2011**	2	1250	4	1825	0.2	25	1620	18	1528	0.2
**R3: May 2011**	14	1707	9	1713	0.7	30	1537	18	1500	0.7
**R4: Aug 2011**	28	1543	30	1450	0.42	41	1178	32	1125	0.42
**R5: Jan 2012**	30	1393	28	14890	0.07	39	1172.	25	1044	0.07

As explained earlier in the data collection methods, these are prices reported by the shop-keeper to the surveyor. Self-reported prices may in some cases suffer from “social desirability bias” and may not reflect true price paid by end patients.

## Discussion

This is the first study to systematically and rigorously measure the availability and retail price of AMFm-subsidized ACT across retail outlets with varying degrees of remoteness. It shows that aggregate availability of AMFm-subsidized ACT increased substantially within 6–8 months of the launch of AMFm in Tanzania, even though some regional differences in availability remained. While this paper does not explore changes in ACT use under the AMFm, it shows that ACT availability in drug shops across two remote regions of Tanzania has increased dramatically under the AMFm and that ACT availability in drug shops farther away from main towns and main roads has also increased significantly. In the first survey round, the average retail price of subsidized ACT was much higher (1,529 TZS or 1.03 USD) than the government recommended price of 1,000 TZS (0.64 USD). However, the price continued to decrease and by the fifth survey round the mean retail price was 1,272 TZS (0.81 USD), with the median price at the recommended 1000 TZS. Similar to availability, there are regional differences in retail prices, with prices in the Rukwa region higher than in Mtwara.

Although initial availability of subsidized ACT was higher in drug shops closer to suppliers or drug shops located proximally to towns, this difference decreased over time and does not appear to be a difference that would persist in the long run. The initial aim of this operational research study was to test a supply-side incentive that could improve availability of AMFm-subsidized ACT in remote shops, if availability in remote shops was lower. The study team decided not to launch an incentive, as availability in remote shops increased much more rapidly than expected.

Significant differences in availability across Rukwa and Mtwara—two very different regions of Tanzania with varying geography, transport/trade linkages and malaria ecology—persisted into the last survey round. Rukwa, which is much farther from Dar es Salaam, has much lower population density than Mtwara, and lower malaria prevalence, had lower availability of subsidized ACT. A larger fraction of the drug shops in Mtwara obtained their supplies directly from Dar es Salaam, whereas in Rukwa they rely on local wholesalers in Sumbawanga or Mbeya. This structural difference in sourcing could explain some of the temporal lag in availability in Rukwa because the local wholesalers could have taken longer to stock and promote subsidized ACT. It is worth noting that while the availability in Rukwa was very low as compared to Mtwara in the first round of analysis, it increased rapidly during the period of the study. This study also finds that prices in Rukwa were significantly higher than Mtwara in the last two survey rounds. The higher prices in Rukwa could be due to the higher cost of transport and distribution to this region compared to Mtwara. Differences in malaria prevalence between the regions could also contribute to differences in availability and prices.

Geographic parameters, particularly measures of distance to large population centers, and distance from major roads, form the basis of most published indices of remoteness. Literature in trade, poverty and medicine [16] consider a purely geographic measure of remoteness, which excludes any consideration of socio-economic status and other demand side factors. The findings of this study suggest that it may be worth exploring more context-specific definitions of remoteness and that understanding socio-economic factors, sourcing patterns and demand creation activities by wholesalers and sub-wholesalers may be key to ensuring more widespread and equitable availability of ACT. Further study should also examine differences between Rukwa and Mtwara using an index like the Rural Access Index (RAI) which measures the number of rural people who live within two kilometers of an all-season road as a proportion of the total rural population [17].

A related study by Cohen *et al.*[9] suggested that existing supply chains for anti-malarials may not reach all individuals in rural regions and special interventions might be needed. The current study shows that existing supply chains are able to serve remote rural drug shops as well. The differences in the findings may stem from the fact that the Cohen *et al.*[9] results were based on a small pilot study using a single wholesaler/distributor whereas the results presented here are from a national roll-out of the AMFm subsidies with multiple wholesalers serving each region. The national scale AMFm is able to take advantage of market competition and economies of scale that were absent or limited in the pilot study. Additionally, the national scale-up of AMFm which was accompanied by nationwide media campaigns to raise awareness about the availability and price of ACT which was not the case in the pilot study in [9].

Availability of child and adolescent doses of AMFm-subsidized ACT was lower as compared to adult doses, possibly due to lower quantities of non-adult packs imported by the national importers/distributors under the AMFm program (Additional file 4: Table C1). It is unclear to what extent the quantities of each pack size of subsidized ACT imported by the national wholesalers/distributors reflected true end patient demand or was the result of other market dynamics.

There are a number of caveats to this study. First, the study was undertaken in only two purposively sampled regions of Tanzania. While the results should have validity outside the regions considered, caution should still be used in directly applying these findings to all other settings. The results may be strongly dependent on socioeconomic factors, malaria treatment-seeking behavior, the structure of the wholesale and retail markets and the structure and performance of the public sector--factors that are often very different between and within countries. Due to logistical and resource constraints, a full-fledged study to capture the stocking of ACT in the public sector, in the two regions, was not undertaken. As a result, any changes in public sector availability were not systematically recorded or analyzed. Additionally, measures of price were self-reported by the ADDO owner or shop attendant.

In summary, this research indicates that the AMFm has significantly increased availability of ACT in Tanzania including in remote drug shops. Even though disparities exist in availability of ACT across the two regions of Tanzania that were a part of the study, these disparities seem to be disappearing over time. The findings show that large inequities in availability of subsidized ACT are only short term and the long run availability of subsidized ACT becomes more equitable both across remote and non-remote drug shops, and across regions. Similar to availability, there are regional differences in retail prices but median retail prices have been decreasing over time. This study does not capture how increased availability translates into increased ACT use, which is the focus of ongoing research in the two regions of this study.

## Abbreviations

ACT: Artemisinin-based combination therapy; ADDO: Accredited drug dispensing outlet; AL: Artemether -lumefantrine; AMFm: Affordable medicines facility for malaria; ASAQ: Artesunate and amodiaquine; GIS: Geographic information system; GPS: Global positioning system; PCA: Principal components analysis; NMCP: National malaria control program; TFDA: Tanzania food and drug authority.

## Competing interests

OS is a member of, and JC receives research support from, the Clinton Health Access Initiative, which is actively supporting efforts to expand funding for and implementation of ACT treatment in the private sector.

## Authors’ contributions

PY, JC, and JA were involved in the conception, design, analysis, data interpretation and writing of the manuscript. SA participated in the design, coordination of the study, analysis, and drafting of the manuscript. PL performed statistical analyses and was involved in drafting the manuscript. OS and JM provided inputs to study design, study context, and drafting of the manuscript. All authors read and approved of the final manuscript.

## Supplementary Material

Additional file 1 **ADDO Characteristics.** Details of ADDOs. Click here for file

Additional file 2 **Non AMFm WHO approved ACT availability.** Details on availability of Non AMFm subsidized but WHO approved ACT. Click here for file

Additional file 3 **Shopkeeper Reported Retail Price.** Details on Shopkeeper Reported Retail Price. Click here for file

Additional file 4 **Tanzania AMFm orders.** Description: Details on AMFM orders in Tanzania. Click here for file
