# Supplementary material for: Trends in availability and prices of subsidized ACT over the first year of the AMFm: evidence from remote regions of Tanzania
Source: Malar J. 2012 Aug 28;11:299. doi: 10.1186/1475-2875-11-299 (PMC3502171; doi:10.1186/1475-2875-11-299)
Supplement: Additional file 1 — ADDO Characteristics. Details of ADDOs. [file 1475-2875-11-299-S1.docx]

| **ADDO Characteristics** | | |
| --- | --- | --- |
| **Table B1:** Percent of Drug Shops with at least one employee who has completed secondary education | | |
|  | Freq | Percent |
| Overall (n=259) | 192 | 74.13% |
| Mtwara (n=112) | 86 | 76.79% |
| Rukwa (n=147) | 106 | 72.11% |

**Table B2**: Percentage of drug shops reporting employees with health qualifications

| **Health Training Type** | **Percent** |
| --- | --- |
| Pharmacist | 86.87% |
| Pharm Tech | 10.81% |
| Pharm Assistant | 0.39% |
| Doctor | 0.77% |
| Dispenser | 20.46% |
| Other Health Training | 11.20% |

**Table B3:** Mean number of persons per Drug Shop that can prescribe or dispense medicines

| **Region** | **Mean** |
| --- | --- |
| Mtwara (n=112) | 1.33 |
| Rukwa (n=147) | 1.45 |

**Table B4:** Percentage of Drug Shops reporting someone attending a health training in the last 12 months

|  | **Freq** | **Percent** |  |  |  |
| --- | --- | --- | --- | --- | --- |
| Overall (n=259) | 86 | 33.20% |  |  |  |
| Mtwara (n=112) | 46 | 41.07% |  |  |  |
| Rukwa (n=147) | 40 | 27.21% |  |  |  |

**Table B5:** Percentage of Health Trainings attended in the last 12 months that were focused on malaria

|  | **Freq** | **Percent** |  |  |  |
| --- | --- | --- | --- | --- | --- |
| Overall (n=86) | 65 | 75.58% |  |  |  |
| Mtwara (n=46) | 36 | 78.26% |  |  |  |
| Rukwa (n=40) | 29 | 72.50% |  |  |  |

**Table B6:** Method of Sourcing Supplies

|  | **All Regions (n=259)** | **Mtwara (n=112)** | **Rukwa (n=147)** |
| --- | --- | --- | --- |
| Someone from the shop goes to the supplier | 89.58% | 88.39% | 90.48% |
| Place order by phone, and supplier delivers directly to shop | 7.34% | 8.04% | 6.80% |
| Place order by phone, supplier delivers it to another location and someone from the shop picks it up | 2.70% | 3.57% | 2.04% |
| Gives money to transporter who goes to the supplier to buy the medicines | 0.39% | - | 0.68% |

**Table B7**: Source of ADDO’s medicine supplies

| Location | Mtwara | Rukwa |  |
| --- | --- | --- | --- |
|  | (n=112) | (n=147) |  |
| own town/village | 8.04% | 19.05% |  |
| Mtwara town | 50% | - |  |
| Masasi town | 9.82% | - |  |
| Dar Es Salaam | 32.14% | 3.40% |  |
| Sumbawanga Town | - | 63.95% |  |
| Mbeya | - | 13.61% |  |
