# Supplementary material for: Trends in availability and prices of subsidized ACT over the first year of the AMFm: evidence from remote regions of Tanzania
Source: Malar J. 2012 Aug 28;11:299. doi: 10.1186/1475-2875-11-299 (PMC3502171; doi:10.1186/1475-2875-11-299)
Supplement: Additional file 2 — Non AMFm WHO approved ACT availability. Details on availability of Non AMFm subsidized but WHO approved ACT. [file 1475-2875-11-299-S2.docx]

| **Increase in Availability of Non AMFM subsidized WHO prequalified ACT** |  |  |  |  |
| --- | --- | --- | --- | --- |
