# Supplementary material for: Trends in availability and prices of subsidized ACT over the first year of the AMFm: evidence from remote regions of Tanzania
Source: Malar J. 2012 Aug 28;11:299. doi: 10.1186/1475-2875-11-299 (PMC3502171; doi:10.1186/1475-2875-11-299)
Supplement: Additional file 3 — Shopkeeper Reported Retail Price. Details on Shopkeeper Reported Retail Price. [file 1475-2875-11-299-S3.docx]

| **A. Shopkeeper Reported Retail Price** | | | | | | | | | | | | | |
| --- | --- | --- | --- | --- | --- | --- | --- | --- | --- | --- | --- | --- | --- |
| **Table A1:** Round 1-Round 5 ACTm Median and Mean Shopkeeper Reported Retail Price by Pack Size | | | | | | | | | | | | | |
|  | | | | **Round 1** | | **Round 2** | | **Round 3** | | **Round 4** | | **Round 5** | |
| **Drug Type** | **ACTm Pack Size** | **N** | **Currency** | **Median** | **Mean** | **Median** | **Mean** | **Median** | **Mean** | **Median** | **Mean** | **Median** | **Mean** |
| Artemether Lumefantrine | 6x1 | (n1=3, n2=14,n3=10, n4=34, n5=73) | **TZS** | 2050 | 2050 | 1000 | 957 | 1000 | 913 | 750 | 794 | 1000 | 896 |
|  |  |  | **USD** | 1.34 | 1.36 | 0.67 | 0.65 | 0.67 | 0.61 | 0.48 | 0.51 | 0.64 | 0.57 |
|  | 6x2 | (n1=2, n2=11, n3=12, n4=24, n5=60) | **TZS** | 750 | 750 | 1000 | 1091 | 1000 | 1283 | 1000 | 825 | 1000 | 962 |
|  |  |  | **USD** | 0.5 | 0.5 | 0.67 | 0.74 | 0.67 | 0.86 | 0.64 | 0.53 | 0.64 | 0.61 |
|  | 6x3 | (n1=0, n2=6 , n3=9, n4=16, n5=55) | **TZS** | -- | -- | 1500 | 1417 | 1500 | 1300 | 1000 | 1006 | 1000 | 1123 |
|  |  |  | **USD** |  |  | 1.01 | 0.96 | 1 | 0.87 | 0.64 | 0.64 | 0.64 | 0.72 |
|  | 6x4 | (n1=28, n2=58, n3=80, n4=139, n5=143) | **TZS** | 1500 | 1529 | 1500 | 1578 | 1500 | 1580 | 1500 | 1299 | 1000 | 1272 |
|  |  |  | **USD** | 1.01 | 1.03 | 1.01 | 1.06 | 1 | 1.06 | 0.96 | 0.83 | 0.64 | 0.81 |
| Artesunate Amodiaquine | 3x1 (25mg/  67.5mg) | (n1=0, n2=0, n3=0, n4=0, n5=1) | **TZS** | -- | -- | -- | -- | -- | -- | -- | -- | 1000 | 1000 |
|  |  |  | **USD** |  |  |  |  |  |  |  |  | 0.64 | 0.64 |
|  | 3x1 (100mg/270mg) | (n1=0, n2=0, n3=0, n4=3, n5=1) | **TZS** | -- | -- | -- | -- | -- | -- | 1000 | 833 | 1000 | 1000 |
|  |  |  | **USD** |  |  |  |  |  |  | 0.64 | 0.53 | 0.64 | 0.64 |
|  | 3x2 (100mg/270mg) | (n1=0, n2=0, n3=0, n4=1, n5=0) | **TZS** | -- | -- | -- | -- | -- | -- | 1000 | 1000 | -- | -- |
|  |  |  | **USD** |  |  |  |  |  |  | 0.64 | 0.64 |  |  |
|  | 12+12 | (n1=0, n2=0, n3=0, n4=0, n5=1) | **TZS** | -- | -- | -- | -- | -- | -- | -- | -- | 1500 | 1500 |
|  |  |  | **USD** |  |  |  |  |  |  |  |  | 0.96 | 0.96 |
