# Supplementary material for: Trends in availability and prices of subsidized ACT over the first year of the AMFm: evidence from remote regions of Tanzania
Source: Malar J. 2012 Aug 28;11:299. doi: 10.1186/1475-2875-11-299 (PMC3502171; doi:10.1186/1475-2875-11-299)
Supplement: Additional file 4 — Tanzania AMFm orders. Description: Details on AMFM orders in Tanzania. [file 1475-2875-11-299-S4.docx]

| **Tanzania AMFm Orders** | | |  | |  |  | |  |  |  |
| --- | --- | --- | --- | --- | --- | --- | --- | --- | --- | --- |
| **Table C1:** Tanzania Private Sector AMFm Orders (August 2010-February 2012 ) | | | | | | | | |  |  |
|  |  |  | |  | |  |  | |  |  |
| **First-Line Buyer Name** | **Manufacturer** | **Drug Type** | | **Dosage Strength** | | **Form** | **Treatment Unit ¹** | | **Sum of Orders Approved Actual** | **Sum of Orders Delivered Actual** |
| ASTRA PHARMA | Ajanta Ltd. | Artemether Lumefantrine | | 20 mg/120 mg | | FDC | 6x1 | | 1,210,000 | 284,960 |
|  |  | Artemether Lumefantrine | | 20 mg/120 mg | | FDC | 6x2 | | 850,000 | 109,890 |
|  |  | Artemether Lumefantrine | | 20 mg/120 mg | | FDC | 6x3 | | 450,000 | 109,980 |
|  |  | Artemether Lumefantrine | | 20 mg/120 mg | | FDC | 6x4 | | 2,025,000 | 1,798,000 |
| JILICHEM TANZANIA LIMITED | Ipca Ltd. | Artemether Lumefantrine | | 20 mg/120 mg | | FDC | 6x1 | | 582,400 | 99,990 |
|  |  | Artemether Lumefantrine | | 20 mg/120 mg | | FDC | 6x2 | | 515,800 | 99,990 |
|  |  | Artemether Lumefantrine | | 20 mg/120 mg | | FDC | 6x3 | | 144,000 | 0 |
|  |  | Artemether Lumefantrine | | 20 mg/120 mg | | FDC | 6x4 | | 1,345,600 | 995,820 |
| PHILLIPS PHARMACEUTICALS | Novartis | Artemether Lumefantrine | | 20 mg/120 mg | | Disp. | 6x1 | | 1,125,840 | 823,440 |
|  |  | Artemether Lumefantrine | | 20 mg/120 mg | | Disp. | 6x2 | | 513,600 | 362,400 |
|  |  | Artemether Lumefantrine | | 20 mg/120 mg | | FDC | 6x3 | | 181,680 | 114,480 |
|  |  | Artemether Lumefantrine | | 20 mg/120 mg | | FDC | 6x4 | | 1,953,270 | 1,761,270 |
| SALAMA PHARMACEUTICALS | Cipla Ltd | Artemether Lumefantrine | | 20 mg/120 mg | | FDC | 6x1 | | 320,000 | 320,000 |
|  |  | Artemether Lumefantrine | | 20 mg/120 mg | | FDC | 6x2 | | 330,000 | 330,000 |
|  |  | Artemether Lumefantrine | | 20 mg/120 mg | | FDC | 6x3 | | 420,000 | 170,000 |
|  |  | Artemether Lumefantrine | | 20 mg/120 mg | | FDC | 6x4 | | 950,000 | 450,000 |
| JD PHARMACY LTD | Africasoins SAS | Artesunate Amodiaquine | | 25 mg/67.5 mg | | FDC | 3x1 | | 90,000 | 75,000 |
|  |  | Artesunate Amodiaquine | | 50 mg/135 mg | | FDC | 3x1 | | 110,000 | 100,000 |
|  |  | Artesunate Amodiaquine | | 100mg/270 mg | | FDC | 3x1 | | 120,000 | 100,000 |
|  |  | Artesunate Amodiaquine | | 100mg/270 mg | | FDC | 3x2 | | 400,000 | 300,000 |
| PHILLIPS PHARMACEUTICALS | Guilin Ltd. | Artesunate Amodiaquine | | 50 mg/150 mg | | Co-b | 3+3 | | 20,000 | 20,000 |
|  |  | Artesunate Amodiaquine | | 50 mg/150 mg | | Co-b | 6+6 | | 20,000 | 20,000 |
|  |  | Artesunate Amodiaquine | | 50 mg/150 mg | | Co-b | 12+12 | | 20,000 | 20,000 |
| **TOTAL** |  |  | |  | |  |  | | **13,697,190** | **8,465,220** |

**Table C2:** Tanzania Public Sector AMFm Orders (August 2010-February 2012)

| **First-Line Buyer Name** | **Manufacturer** | **Drug Type** | **Dosage Strength** | **Form** | **Treatment Unit ¹** | **Sum of Orders Approved Actual** | **Sum of Orders Delivered Actual** |
| --- | --- | --- | --- | --- | --- | --- | --- |
| MEDICAL STORES DEPARTMENT | Novartis | Artemether Lumefantrine | 20 mg/120 mg | Disp. | 6x1 | 2,244,510 | 2,244,510 |
|  | Novartis | Artemether Lumefantrine | 20 mg/120 mg | Disp. | 6x2 | 944,010 | 944,010 |
|  | Novartis | Artemether Lumefantrine | 20 mg/120 mg | FDC | 6x3 | 510,570 | 510,390 |
|  | Novartis | Artemether Lumefantrine | 20 mg/120 mg | FDC | 6x4 | 1,218,690 | 1,218,690 |
| **TOTAL** |  |  |  |  |  | **4,917,780** | **4,917,600** |
